# Image guided dose escalated prostate radiotherapy: still room to improve

**DOI:** 10.1186/1748-717X-4-50

**Published:** 2009-11-03

**Authors:** Jarad M Martin, Andrew Bayley, Robert Bristow, Peter Chung, Mary Gospodarowicz, Cynthia Menard, Michael Milosevic, Tara Rosewall, Padraig R Warde, Charles N Catton

**Affiliations:** 1 Princess Margaret Hospital, Department of Radiation Oncology, Princess Margaret Hospital, University of Toronto, Toronto, Ontario, Canada; 2 Cancer Care Services, Royal Brisbane and Women's Hospital, Herston, Queensland, Australia

## Abstract

**Background:**

Prostate radiotherapy (RT) dose escalation has been reported to result in improved biochemical control at the cost of greater late toxicity. We report on the application of 79.8 Gy in 42 fractions of prostate image guided RT (IGRT). The primary objective was to assess 5-year biochemical control and potential prognostic factors by the Phoenix definition. Secondary endpoints included acute and late toxicity by the Radiotherapy Oncology Group (RTOG) scoring scales.

**Methods:**

From October/2001 and June/2003, 259 men were treated with at least 2-years follow-up. 59 patients had low, 163 intermediate and 37 high risk disease. 43 had adjuvant hormonal therapy (HT), mostly for high- or multiple risk factor intermediate-risk disease (n = 25). They received either 3-dimensional conformal RT (3DCRT, n = 226) or intensity modulated RT (IMRT) including daily on-line IGRT with intraprostatic fiducial markers.

**Results:**

Median follow-up was 67.8 months (range 24.4-84.7). There was no severe (grade 3-4) acute toxicity, and grade 2 acute gastrointestinal (GI) toxicity was unusual (10.1%). The 5-year incidence of grade 2-3 late GI and genitourinary (GU) toxicity was 13.7% and 12.1%, with corresponding grade 3 figures of 3.5% and 2.0% respectively. HT had an association with an increased risk of grade 2-3 late GI toxicity (11% v 21%, p = 0.018). Using the Phoenix definition for biochemical failure, the 5 year-bNED is 88.4%, 76.5% and 77.9% for low, intermediate and high risk patients respectively. On univariate analysis, T-category and Gleason grade correlated with Phoenix bNED (p = 0.006 and 0.039 respectively). Hormonal therapy was not a significant prognostic factor on uni- or multi-variate analysis. Men with positive prostate biopsies following RT had a lower chance of bNED at 5 years (34.4% v 64.3%; p = 0.147).

**Conclusion:**

IGRT to 79.8 Gy results in favourable rates of late toxicity compared with published non-IGRT treated cohorts. Future avenues of investigation for toxicity reduction include IMRT, margin reduction, and dose modulation targeted to sites of disease burden. Further work is required to maximize efficacy beyond that achieved through radiation dose escalation alone.

## Background

Prostate cancer is the most commonly cancer diagnosis in Canadian men. External beam radiation therapy (EBRT) has an established role in the management of localized prostate cancer, although historical series have shown relatively poor outcomes and high toxicity [1]. This led to the investigation of various adjuncts to EBRT to improve the therapeutic ratio, with adjuvant hormonal deprivation, in particular, proving successful for men with high risk disease [2].

A steep radiation dose-response curve has been postulated, and investigated in 3 reported randomized studies to date using doses of between 78-79.2 Gy in the dose escalated arm [3-6]. All of these studies have shown an improvement in biochemical outcomes, although often only in particular risk stratification subgroups. The study with the most mature follow-up is also beginning to show an advantage in clinical endpoints [4].

All of the dose escalation studies have shown an increase in late toxicity, and various strategies have been implemented to help minimize this. Firstly, treatment based on 2-dimensional planning has been proven more toxic than with 3-dimensional conformal radiotherapy (3DCRT), with the latter widely practiced alongside intensity modulated radiotherapy (IMRT) [7-9]. Another is the recognition of the dose sensitivity of the rectum, and the routine use of dose constraints for this and other critical structures [10]. Soft tissue image guided radiotherapy (IGRT) has also been widely introduced to help deal with interfraction organ motion [11]. Although a variety of approaches are in use, intraprostatic gold fiducials are the most widespread in clinical practice [12].

After investigating the efficacy of 75.6 Gy in 42 fractions, the Princess Margaret Hospital continued to utilize all of the above approaches prior to escalating prostate dose to 79.8 Gy in 42 fractions [13]. Here we present 5-year efficacy and toxicity outcome data for this treatment practice.

## Methods

### Study Design

Retrospective analysis of a prospectively maintained institutional prostate cancer RT database. The first 302 consecutive men treated with curative intent with RT at PMH following the implementation of 79.8 Gy in October 2001 as the standard treatment approach for localized disease formed the study cohort. This project has University Health Network institutional human research ethics committee approval (REB 08-0473-CE).

### Study population

Eligible patients had biopsy confirmed adenocarcinoma of the prostate with clinical stage T1-3N0 M0. All patient details were independently verified, corrected and updated by two different radiation oncologists. Patient enrolled on a concurrent randomized trial receiving 5 months of bicalutamide in the experimental arm were excluded from the study cohort.

Staging CT and bone scans were not routinely performed for those with low and intermediate risk disease. Patients with less than 2 years of follow-up data available were excluded to reduce bias in under-reporting of toxicity due to an insufficient period of observation.

### Treatment Planning

The radiotherapy planning and treatment delivery has been previously reported [13]. All patients had 3 gold fiducial markers (1 mm diameter by 5 mm long) inserted transrectally under ultrasound guidance into the prostate at base, apex and midgland. Patients were immobilized in the supine position with a VacLoc cradle extending from hip to thigh and leg stocks to immobilize knees. Patients were given specific instructions regarding daily preparation to include a comfortably full bladder via consumption of 500 mL of water within 1 hour of planning and an empty rectum via the use of milk of magnesia for one week beforehand. This approach was continued throughout treatment as well. If the rectum was distended with gas or feces on the scout CT, the patient was asked to evacuate prior to proceeding with the full scan. Axial images were obtained at 5 mm through the pelvis and at 2 mm intervals through the prostate, using a helical scanner.

Contouring structures were delineated using ICRU-62 convention. The GTV was the prostate. The CTV was the same as GTV, except for men with a predicted risk of seminal vesicle (SV) involvement of greater than 15%, in which case the proximal 10 mm of the SV were included in the CTV [14]. The PTV was CTV plus a uniform 10 mm margin except posteriorly where 7 mm was used. No one received elective pelvic lymph node irradiation. Femoral heads, bladder walls and rectal walls were contoured throughout the treatment volume (18 mm superior and/or inferior from limits of the CTV as appropriate).

### Radiation prescription

The radiation prescription dose was 79.8 Gy in 42 fractions given in 5 fractions per week. Doses were prescribed to the ICRU reference point, with the PTV contained within the 95% isodose line. IGRT verification dose was incorporated into the plan. Critical structure dose constraints used are shown in table 1. The standard treatment approach incorporated a six-field class solution with gantry angles of 60, 90, 120, 240, 270 and 300 degrees, although these angles could be modified to optimize treatment plans. An anterior field (0 degrees) was used for orthogonal imaging of the fiducial markers. If dose constraints were exceeded, an IMRT inverse plan was used. In all cases a coplanar beam arrangement was applied with 6 MV for IMRT plans, and 6 or 18 MV photons for 3DCRT plans. Helios inverse treatment planning module within CADplan 6.27 was used for IMRT planning.

**Table 1:** Dose constraints for patients treated to 79.8 Gy for localized prostate cancer.

PTV	ICRU point dose 79.8 Gy
	Dose to 99% of volume (D99): 75.6 Gy
	Maximum: 81.4 Gy ≤ contiguous 2 cm^3^.
	
Bladder wall	70% volume receives less than 70 Gy.
	50% volume receives less than 55 Gy.
	
Rectal wall	70% volume receives less than 70 Gy;
	50% volume receives less than 55 Gy.
	
Right/Left femoral head	Maximum dose 55 Gy

### Treatment delivery

Field placement verification was done by means of daily electronic portal imaging of an antero-posterior and a lateral field using an amorphous Silicon array. Fiducial marker centre of mass was matched electronically to reference images by the treating technologist, and deviations of more than 3 mm in any orthogonal plane corrected on-line prior to treatment being delivered.

### Follow-Up

Weekly review of patients was conducted by the radiation oncologist to manage any acute reactions. Acute symptoms were prospectively scored by treating Therapists and recorded in the electronic patient record using RTOG criteria [15]. There was no formal follow-up procedure, but follow-up policy included appointments between 4-12 weeks following the completion of treatment to assess toxicity resolution, then every six months for assessment of late toxicity, clinical and biochemical control.

### Endpoints

The primary endpoint was 5-year biochemical no evidence of disease (bNED) according to the nadir + 2 definition [16]. The bNED using the previous ASTRO definition of three consecutive rises backdated is also reported to allow intercomparison with older literature. Instigation of salvage therapies and evidence of clinical disease progression prior to a PSA rise where also counted as a failure. For the ASTRO definition, hormone use lead to patients being excluded from bNED analysis. Peak physician-assessed acute and late toxicity was graded according to the RTOG criteria for actuarial reporting. Clinical relapse in lymph-nodes or bones was also recorded, as was instigation of salvage interventions (local, hormonal or chemotherapeutic). Any metachronous malignancies were recorded, as were any deaths and their causes.

### Statistics

Patients were censored either at the time of an event, or the time of last review, whichever occurred first. Kaplan Meier curves for biochemical control using the two failure definitions were generated using SPSS v17.0. Univariate analyses for potential prognostic factors (age, PSA, Gleason score, T-category, risk stratification, hormonal use) were performed for the nadir + 2 biochemical failure definition. Multi-variate analyses were performed using a Cox-Regression model. Chi-squared tests were performed to assess for independence in categorical data. A log-rank p-value of less than 0.05 was considered significant.

## Results

22 men were excluded with less than 24 months of follow-up, and a further 21 men were excluded as they received hormonal therapy on the experimental arm of a concurrent clinical trial. Overall 259 patients were identified who fitted the inclusion criteria. They commenced radiotherapy between October 2001 and June 2003. Baseline characteristics of patients treated according to protocol, including risk stratification as per Canadian Consensus Guidelines, are shown in table 2[17]. Median follow-up for all patients is 67.8 months (range 24.4-84.7).

**Table 2:** Patient characteristics.

Age	
Median (range)	71 Years (45 - 84)
Clinical T-category	
T1b	1
T1c	83 (T1 32%)
T2a	125
T2b	15 (T2 65%)
T2c	28
T3a	2
T3b	2 (T3 2%)
TX	3
Gleason score	
5-6	96 (37%)
7	141 (55%)
8-10	21 (8%)
Initial PSA (ng/mL)	
Median (range)	7.6 (0.26-51.4)
Risk stratification	
Low	59 (22%)
Intermediate	163 (63%)
High	37 (14%)
Radiotherapy Treatment	
3-Dimensional Conformal	226 (87%)33
IMRT	(13%)

### Acute toxicity

The worst RTOG acute toxicity scores are shown in table 3. No instances of grade 3-4 toxicity were observed during treatment.

**Table 3:** Worst Radiation Therapy Oncology Group (RTOG) acute effects score during treatment.

	**Grade 0**	**Grade 1**	**Grade 2**	**Grade 3**
Gastrointestinal (n = 257)	39.0%	50.9%	10.1%	0.0%
Genitourinary (n = 256)	16.3%	50.4%	33.3%	0.0%

### Late toxicity

**Figure 1:**
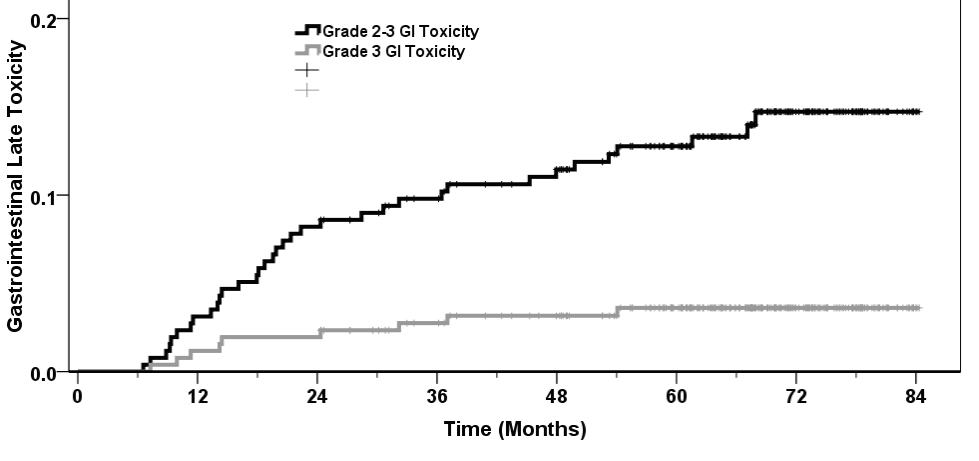
**Actuarial RTOG grade 2-3 late gastrointestinal toxicity for 259 men treated for localized prostate cancer with IG-3DCRT to 79.8 Gy.** Minimum followup is 2 years.

**Figure 2:**
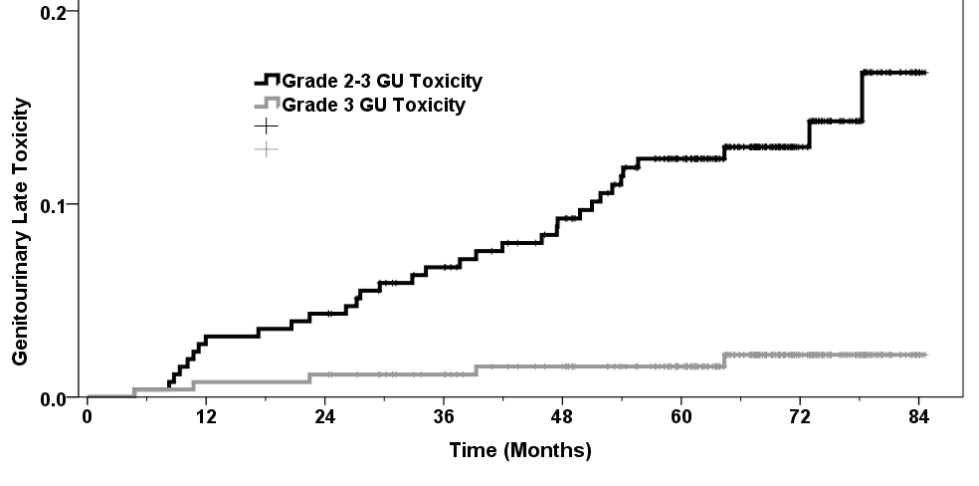
**Actuarial RTOG grade 2-3 late genitourinary toxicity for 259 men treated for localized prostate cancer with IG-3DCRT to 79.8 Gy.** Minimum followup is 2 years.

No RTOG grade 4-5 late toxicity was recorded. Table 4 shows late toxicity at the time of last assessment. The actuarial rate of late gastrointestinal (GI) toxicity grade 2-3 was 13.7% at 5 years, and the corresponding figure for genitourinary (GU) toxicity was 12.1%. The comparable figures for grade 3 toxicity were 3.5% (GI) and 2.0% (GU) respectively. These data are summarized in Figures 1 and 2. Nine men had a Grade 3 late GI toxicity event after a median of 14 months (range 7 - 54) usually consisting of heavy bleeding and managed either with hyperbaric oxygen, laser photocoagulation or observation. Five men have had a grade 3 late GU toxicity event at 5, 11, 22, 39, and 64 months consisting of hematuria (3), stricture (1) and obstruction (1). One man suffered both intractable grade 3 GI and GU complications. Of the 13 patients who experienced a grade 3 late toxicity with subsequent follow-up available, 7 had either grade 0 or 1 toxicities recorded at the time of their last assessment.

**Table 4:** Radiation Therapy Oncology Group (RTOG) late effects score at most recent followup.

	**Grade 0**	**Grade 1**	**Grade 2**	**Grade 3**
Gastrointestinal (n = 256)	91.8%	3.9%	3.1%	1.2%
Genitourinary (n = 257)	83.3%	8.2%	7.4%	1.2%

### Adjuvant Hormonal therapy

43 men (16.8%) received some form of hormonal therapy (HT), of whom 6 were prescribed an anti-androgen and 37 (14.3%) were prescribed a LHRH agonist. Most of these men had either high-risk or multiple risk factor intermediate-risk disease (n = 25). Courses were usually short (<6 months) for low-intermediate risk men, and longer (24-36 months) for high risk men. 13.6% of low, 11.0% of intermediate and 45.9% of high risk patients received HT (Chi-Squared = 27, p < 0.001). Hormone therapy had a borderline association with increasing G2-3 peak late GI toxicity (5-year actuarial rates 11% v 22%; p = 0.025, HR = 2.27 [95% CI 1.11-4.63]), but not G2-3 late GU toxicity (5-year actuarial rates 14% v 10%; p = 0.78, HR = 0.87 [95% CI 0.34-2.27]).

### Biochemical Response

**Figure 3:**
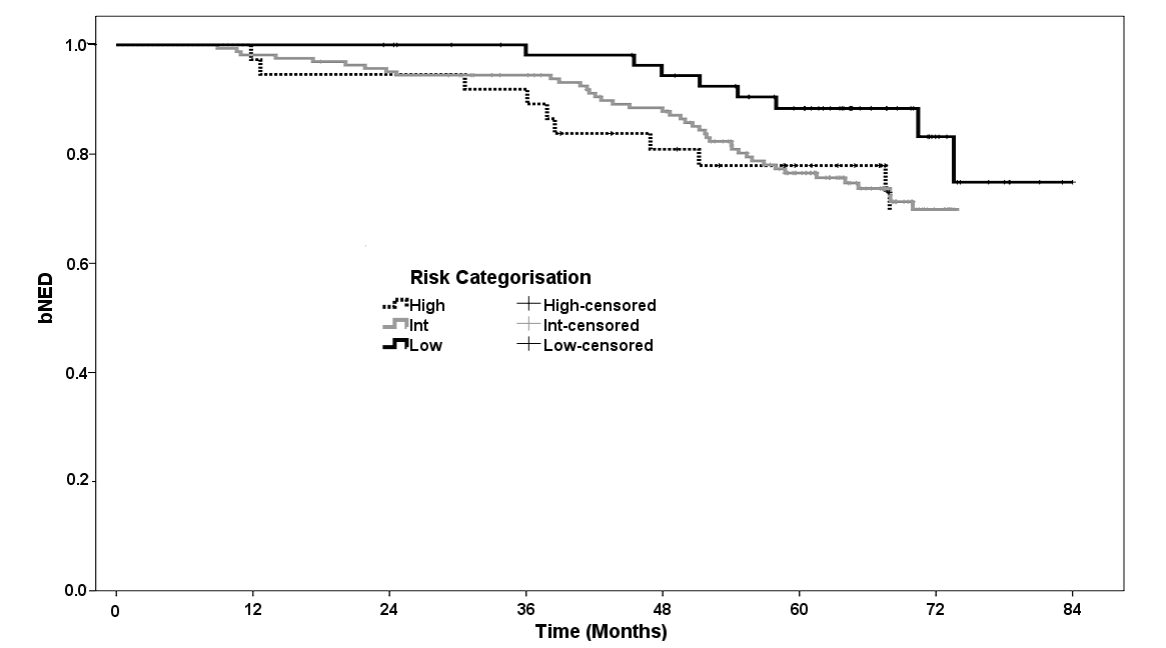
**Biochemical control by nadir + 2 definition and risk stratification for 259 men treated for localized prostate cancer with IG-3DCRT to 79.8 Gy.** Minimum followup is 2 years.

Using the nadir +2 definition for biochemical failure, the rate of biochemical control for the entire cohort at 5 years is 79.4%. By the ASTRO definition (3 consecutive rises, backdated) the corresponding figure is 67.9%. Using the ASTRO definition for biochemical failure, the rate of biochemical control at 5 years were 78.2%, 65.2% and 62.7% for low, intermediate and high risk patients respectively. Figure 3 shows the Kaplan-Meier plot for biochemical control by the nadir + 2 definition. By low, intermediate and high risk categorization, the 5 year biochemical control rates by the nadir + 2 definition are 88.4%, 76.5% and 77.9% respectively (log-rank p = 0.14). Other univariate analyses of biochemical outcome are presented in table 5. On pair-wise comparisons, T-category (T2b-T2c v T1b-T2a) and Gleason score (Gleason 7 v 5-6) were both statistically significant (HR: 2.48; p = 0.002 and HR: 1.73; p = 0.058 respectively). On multivariate analysis using a Cox Regression model and entering PSA (p = 0.42) and age (p = 0.50) as continuous variables, and risk categorization (p = 0.61), T-category (p = 0.04), Gleason score (p = 0.53) and hormone use (p = 0.96) as categorical variables, only T-category displays marginal significance (table 6). Looking specifically at the 141 men with Gleason 7 disease, 99 were pattern 3+4 with the remaining 41 pattern 4+3, with 5 year bNED rates of 73.0% and 76.7% respectively (p = 0.64).

**Table 5:** Univariate analysis of potential prognostic factors for 5-year nadir + 2 biochemical outcome.

**Risk Factor**	**Number**	**Nadir +2** **5-year bNED**	**p-value**	**HR (95%CI)**
PSA				
<10	180	74.4%		
10-20	63	79.4%		
>20	16	75%		
Continuous variable			0.85	1.00 (0.96-1.03)
				
Gleason grade				
5-6	96	82.3%	0.15	(reference)
7	141	71.6%	0.06	1.73 (0.98-3.05)
8-10	21	71.4%	0.21	1.81 (0.71-4.59)
				
T-category				
T1b-T2a	209	83.0%	0.006	(reference)
T2b-T2c	43	64.9%	0.002	2.48 (1.42-4.36)
T3a-TX	7	68.6%	0.56	1.56 (0.37-6.30)
				
Risk Grouping				
Low	59	86.4%	0.14	1 (reference)
Intermediate	163	73%	0.06	2.04 (0.96-4.34)
High	37	70.3%	0.07	2.33 (0.94-5.78)
				
Adjuvant Hormone				
No	165	76.4%	0.52	1 (reference)
Yes	31	72.1%	0.66	0.81 (0.43-1.53)
				
Age				
Continuous variable			0.66	1.01(0.97-1.05)

**Table 6:** Multivariate analysis of prognostic factors for 5-year nadir +2 biochemical outcome in a Cox Regression model.

**Risk Factor**	**Number**	**Nadir +2** **5-year bNED**	**p-value**	**HR (95%CI)**
PSA				
<10	108	72.3%		1 (reference)
10-20	55	86.3%		
Continuous variable			0.62	0.98 (0.91-1.06)
				
Gleason grade				
5-6	31	83.4%		
7	132	75.7%	0.94	0.94 (0.47-2.03)
				
T-category				
T1b-T2a	128	79.5%	0.04	1 (reference)
T2b-T2c	35	64.3%		1.93 (1.01-3.69)
				
Adjuvant Hormone				
No	145	77.5%		1 (reference)
Yes	18	69.3%	0.41	1.44 (0.61-3.42)
				
Age				
Continuous variable			0.57	1.02 (0.96-1.07)

PSA and age are entered as continuous variables

### Intermediate Risk Biochemical Response

Further analyses were performed looked specifically at the subgroup of men with intermediate risk disease using the nadir + 2 biochemical failure definition, presented in table 6. For the 131 men with intermediate risk and Gleason 7 disease, 95 were pattern 3+4 with the remaining 36 being pattern 4+3. Once again, there was no significant difference between the two Gleason 7 subgroups for 5-year bNED (73.2% and 81.3%; p = 0.33). Looking at the number of intermediate risk factors (ie category T2b-c, PSA 10-20 and Gleason 7), men with 1 (n = 107) or 2 (n = 53) such factors had no significant differences in 5-year bNED (76.9% and 76.2% respectively; p = 0.91). On multivariate modeling none of age (p = 0.67), Initial PSA (p = 0.64), T-category (p = 0.07), Gleason grade (p = 0.97), or hormone use (p = 0.48) were significant prognostic factors.

### Local Control and Salvage

26 men had prostate biopsies performed a median of 47 months (range 14.5 - 69.8) following the conclusion of radiotherapy, of whom 12 were positive for disease with the remainder negative or showing radiation effect. A chi-squared test for independence between having a biopsy and experiencing a nadir + 2 failure was significant (p < 0.001), suggesting an association between the two events. Indeed, 4 of the men with a nadir + 2 event, had their prostate biopsy subsequent to this. Men with positive biopsies had a lower chance of remaining bNED at 5 years (34.4% v 64.3%; p = 0.147).

Photodynamic therapy (PDT) was delivered for two men with presumed isolated local relapse with negative imaging and positive prostate biopsies. A further two men had either cryotherapy, or a radical prostatectomy for local salvage. All but one of these four men had a rising PSA on subsequent follow-up.

### Distant Disease and Survival

Six men have developed positive bone scans, four pelvic lymphadenopathy, and one both boney and cerebral metastatic disease. 39 patients have being commenced on salvage hormonal therapy, generally at a relatively early state of biochemical relapse (median PSA = 7.1 at initiation), usually in a continuous rather than intermittent fashion, and all but 2 on an LHRH agonist in the first instance. Four have progressed to become hormone refractory, 3 have received docetaxol, and the other mitoxantrone. 20 men have experienced a metachronous malignancy, with the most common primary sites being colorectal (n = 8), bladder (n = 3), lung (n = 2) and stomach (n = 2). One man progressed through hormonal therapy and chemotherapy, and died with brain metastases, presumed due to prostate cancer, 25 months after commencing RT. Nine further men have died, however none of these were suffering biochemical relapse at this time of last follow-up.

## Discussion

Our experience shows that dose escalated RT (DERT) to a dose of 79.8 Gy can be safely delivered using 3DCRT and IGRT. Our 5-year bNED by the nadir + 2 definition was 79.4% (95% CI 74.1 - 84.6), which approximates the corresponding figure of 85% reported from the 78 Gy arm of the MD Anderson Cancer Centre (MDACC) randomized trial [4]. Our ASTRO outcomes are an improvement over our previously reported results of treating to 75.6 Gy (5 yr bNED 55% v 67.9%) [13], and the Memorial Sloan Kettering results using the same dose for high/unfavourable risk patients (5 yr bNED 63% v 43%) [18]. It is difficult to know if this is a function of radiation dose escalation, heterogeneity within the high risk patient groups, differential hormone administration, or a combination of such factors.

The MDACC randomized trial had a higher proportion of high risk patients than in our series, no image guidance, and CT-planning only following the 4-field phase 1 RT. Hence, the slightly lower bNED in our series is unexpected. Although larger field RT including irradiation of the seminal vesicles have yet to be prospectively validated as improving disease control outcomes, there is some potential that these larger RT volumes may have had a beneficial impact in the MDACC series. Our series used a nomogram cut-off value of 15% prior to inclusion of the seminal vesicles in the CTV. Potentially, similar methods may be helpful in determining the risk of extra-capsular extension and lymph node involvement, although the selection of cut-off values in each of these respects will be empirical in the absence of prospective data. In the era of CTV-PTV margin reduction to reflect the improved understanding and management of organ motion, it remains important to reflect on the Prostate-CTV expansion. The risk of extracapsular disease, subclinical seminal vesicle and pelvic nodal involvement should be considered when the CTV is delineated.

Local relapses were identified on post-treatment biopsy, suggesting that despite more accurate radiation delivery of a higher dose some tumour clonogens will survive. One option is to continue escalating the radiation dose, as has been explored at Memorial Sloan Kettering Cancer Center [19]. Another promising avenue is to exploit the likely fraction sensitivity of prostate cancer [20]. Several centres have recently been investigating the feasibility of hypofractionation [21-25]. Our own experience with 60 Gy delivered in 3 Gy fractions over 4 weeks utilizing IGRT and IMRT is now being explored in a randomized trial compared with a 78 Gy in 2 Gy fraction standard arm (PROFIT - Prostate Fractionated Irradiation Trial)[26] A further strategy is to identify and direct additional treatment at the dominant prostate lesion [27].

Additional follow-up will be necessary to determine the effect of DERT and IGRT on important clinical endpoints such as local, nodal or boney relapse as well as prostate cancer specific survival (PCSS). Only after a median follow-up of 8.7 years did any advantage for such failure endpoints appear for the MDACC RCT, which is now beginning to suggest a PCSS advantage [4]. The advent of effective systemic therapy for hormone refractory prostate cancer, with the potential of others under investigation, may dilute survival impacts of DERT compared with historical cohorts [28,29].

Severe late rectal toxicity was unusual in our cohort, with grade 3 reactions reported in 3.5% of patients. The Cleveland Clinic used ultrasound based IGRT with intensity modulated RT (IMRT) in a hypofractionated manner to a dose of 70 Gy in 28 fractions, and reported a corresponding figure of 2% [22]. PMH has a low threshold for intervention for men with rectal bleeding, which may contribute in differences between series. Cleveland clinic has also shown that IGRT removes any prognostic impact of rectal filling at the time of treatment planning on efficacy and rectal toxicity [30,31]. On the basis of interfraction motion studies, IGRT has become the standard of care, and it is pleasing to see our results supporting such theoretical advantages [11].

The dose escalation randomized trials reported rate of grade 2 or greater late toxicity in some detail [3,4]. For the Dutch trial at the 7 year time-point, there was no difference in late grade 2+ GU late toxicity between the 68 Gy and 78 Gy arms (41% v 40%, p = NS), but a significant worsening of grade 2+ GI late toxicity (25% v 35%, p = 0.04)[3] Similarly, the MDACC trial comparing 70 Gy with 78 Gy showed similar trends in 10 year GU (8% v 13%, p = NS) and GI (13% v 26%, p = 0.013) grade 2+ late toxicity [4]. Allowing for differences in duration of follow-up, the corresponding GI and GU figures from the current series of 12.6% and 12.1% compare favourably with the standard arms of these trials. Although there is potential for under-reporting of late toxicity when data is collected retrospectively, moderate to severe toxicity requiring interventions is less likely to be missed than grade 1 toxicities. It is therefore plausible that newer technologies have allowed the delivery of an extra 8-10 Gy without an increase in toxicity in moderate to severe late rectal toxicity. Both of these randomized studies incorporated some 2-dimensional planning, as well as no IGRT. It would appear that IGRT using either ultrasound or gold fiducials plus standardized 3DCRT planning with relatively conservative DVH constraints can reduce toxicity markedly.

Although there is some evidence that the superior dose-distributions achievable with IMRT offers further a reduction in rectal toxicity compared with 3DCRT, such series usually do not utilise IGRT [7,32]. IMRT was used infrequently in the current series, and given the approximate halving in the rate of Grade 2-3 rectal toxicity in the Cleveland clinic IGRT cohort compared with our own, it is possible that IMRT used in the Cleveland experience could be responsible for some of this improvement. However, the use of a smaller 4-8 mm non-uniform CTV-PTV expansion at that institution may also have contributed to this result. Although there is a suggestion from our series of an association between the use of hormonal therapy and late GI toxicity, this may be due to confounding factors such as larger prostate volumes in men managed with cytoreductive hormonal therapy. Further work in this area is needed to clarify whether such a relationship between hormonal therapy and toxicity exists.

Moderate grade 2 bladder toxicity in the form of irritation or slight hematuria occurred in approximately 12% of our patients during follow-up. One must note that in general urinary toxicity was not enhanced by the delivery of higher doses of radiation in the randomized trials. However, the use of IGRT would ensure that the prostatic urethra receives full dose throughout the treatment course, and therefore one might expect similar toxicity levels as reported in the randomized trials of between 17-41%. This may be part of the reason why bladder DVH constraints have generally correlated relatively poorly with late toxicity [33].

Although toxicities can be self limiting, a focus of further work should be to try to minimize late moderate urinary toxicity. Objective scoring and management of lower urinary tract symptoms prior to treatment may be helpful, with cytoscopic assessment and intervention warranting consideration for men with poor premorbid function. Urethral sparing approaches may be technically feasible, but raises the concern of underdosing malignant tissue which can occur in a peri-urethral distribution as well as difficulties in visualizing the urethral on a daily basis. Greater education regarding bladder base anatomy on CT and MRI may assist with dose reduction to the bladder trigone. It is likely that a combination of such approaches will be required.

There is little consensus on the optimal margin expansion from CTV to PTV. The main contributors to this are interfraction motion, intrafraction motion, and contouring variation. The use of IGRT with a zero mm action threshold should address the first issue. Recent work to quantify intrafraction motion suggests that 2 mm will account for this in the majority of patients for the majority of fractions [34-36]. The use of real time tracking could potentially reduce this further. Contouring variation can be minimized with education and MRI fusion to some extent [37]. Overall the current trend would be to reduce margins to between 5-7 mm, which would be expected to further reduce rates of late rectal toxicity, although mature follow-up of current cohorts will be needed to confirm this.

We were not able to show any advantage for the small number of selected men with intermediate and high risk disease who received adjuvant hormone therapy in the current series, and one area of controversy remains the optimal manner to integrate hormonal therapies (HT) with DERT for men with intermediate and high risk disease [24]. The picture becomes less clear in the era of PSA screening and Gleason grade migration. Although trials are currently underway to address the additive advantage of HT with DERT as standard treatment, such results are pending. In the meantime, selection of men with high-intermediate risk prostate cancer for a short course of HT will be empirical, with a number of suggested approaches including nomograms, Gleason 4+3 disease, PSA>15, and 2-3 intermediate risk factors [38,39]. What is clear is that in the absence of definitive evidence of benefit in this patient subgroup, that toxicities must be discussed with prospective patients, and their involvement is crucial in the decision making process.

Our cohort was predominantly made up of men with low-intermediate risk disease, and did not have a large number of biochemical failures. Hence it is difficult to define risk factors in a relatively homogeneous cohort with good outcomes. The predictive power of positive biopsies has been previously reported, [13] although the current series suggests a systematic bias in clinical practice to biopsy men with a rising PSA. The lack of prognostic significance for the different dominant Gleason 7 patterns may reflect the impact of Gleason grade migration, redefinition of overall Gleason scoring from biopsies and the subsequent 'Will Rogers Effect' [40].

## Conclusion

Dose escalated radiotherapy using predominately 3D conformal approaches and IGRT to a dose of 79.8 Gy is feasible and leads to good rates of biochemical control. Improved CTV definition to address extracapsular extension, seminal vesicle and pelvic nodal occult disease remains an avenue for investigation. Significant rectal and bladder toxicity is unusual, but could potentially be reduced with the routine use of IMRT and a smaller CTV-PTV margin expansion. Further work is needed to optimize total dose, dose per fraction, integration with hormonal therapy, and intra-prostate tumour targeting.

## Conflict of interests

The authors declare that they have no competing interests.

## Authors' contributions

Study design, patient contribution, manuscript review: AB, RGB, PC, MG, CM, MM, TR, PRW, CNC. Data collection: JMM, CNC. Data analysis, manuscript preparation: JMM. All authors reviewed and approved the final manuscript.
