# Correction: Image guided dose escalated prostate radiotherapy: still room to improve

**DOI:** 10.1186/1748-717X-4-65

**Published:** 2009-12-16

**Authors:** Jarad M Martin, Andrew Bayley, Robert Bristow, Peter Chung, Mary Gospodarowicz, Cynthia Menard, Michael Milosevic, Tara Rosewall, Padraig R Warde, Charles N Catton

**Affiliations:** 1 Princess Margaret Hospital, Department of Radiation Oncology, Princess Margaret Hospital, University of Toronto, Toronto, Ontario, Canada; 2 Cancer Care Services, Royal Brisbane and Women's Hospital, Herston, Queensland, Australia

## Abstract

We regret to report that a proofreading error caused an incorrect legend and description of the contents of table 6 to appear in our original publication of this work. The correct description of table 6 is: Univariate analysis of prognostic factors for 5-year nadir + 2 biochemical outcome for men presenting with intermediate risk factors.

## Correction

We regret to report that a proofreading error caused an incorrect legend and description of the contents of table 6 to appear in our original publication of this work [1]. The correct description of table 6 is: Univariate analysis of prognostic factors for 5-year nadir + 2 biochemical outcome for men presenting with intermediate risk factors.
